# clinical characteristsics of adolescents and adults with sickle cell disease and barriers to transition to adult care at Mulago Hospital Uganda; A mixed methods study

**DOI:** 10.21203/rs.3.rs-6234847/v1

**Published:** 2025-05-22

**Authors:** Racheal Owomuhangi, Charles Karamagi, Grace Ndeezi, Japheth Kwiringira, Deogratias Munube, Sarah Kiguli, Robert Opika Opoka, Ruth Namazzi

## Abstract

**Background** Over the past six decades, advancements in medical care have significantly enhanced outcomes for individuals with sickle cell disease (SCD), with studies demonstrating increased survival rates into adulthood. A smooth transition from pediatric to adult care is essential for maintaining positive health outcomes. Nevertheless, healthcare systems have struggled to adapt, leaving gaps in support for the expanding adult SCD Population. Mulago Pediatric Sickle Cell Clinic has faced multiple challenges with the transition to adult care that are not well documented. The objective of this study was to describe the clinical characteristics of adolescents and adults with sickle cell disease and barriers to adult care at Mulago Hospital. **Methods** This was a mixed method cross-sectional study with both qualitative and quantitative data collection methods conducted among patients attending the pediatric sickle cell clinic at Mulago Hospital, their caregivers, and health care workers. A registry and medical records review was done to obtain data for the quantitative arm. The qualitative component consisted of 30 in-depth interviews involving patients and caregivers and 10 key informant interviews with healthcare workers. Quantitative data was coded and entered into Epidata version 4.6 and then exported to STATA 14 for analysis. Qualitative data was analyzed using the content thematic approach. **Results** The proportion of patients aged 14 years and above still attending the pediatric clinic was 21.6%. Barriers to the transition of care as expressed by caregivers and patients were limited knowledge of transition, attachment to their pediatric careers, and negative experiences in the adult clinics. Health care system barriers included poorly organized adult clinics with few working days compared to the pediatric clinic that operates daily. This was compounded by a lack of policies and guidelines on transition, inadequate human resources, and limited access to the essential drugs in the adult clinics. **Conclusions and recommendations** There is still a large proportion of adolescents and young adults still attending the pediatric sickle cell clinic and barriers to transition were not only sociodemographic but also psychosocial and health system related. There is a need for better planning and preparation with better patient-centred interventions to improve transition.

## Background

Sickle cell disease (SCD) is an inherited blood disorder caused by a mutation in the hemoglobin gene, predominantly affecting individuals of sub-Saharan African and South Asian descent. The condition leads to abnormally shaped red blood cells, resulting in chronic hemolytic anemia and Vaso-occlusive crises due to blocked blood flow, which can cause tissue damage and organ dysfunction (1).

Globally, approximately 4.4 million people have sickle cell anemia, while 43 million carry the sickle cell trait, with 75% of cases concentrated in Africa (2, 3). In Uganda, the sickle cell trait prevalence is 13.3%, and sickle cell anemia affects 0.8% of the population, with the highest prevalence observed in the east-central region (4).

Over the past six decades, survival rates for SCD patients have significantly improved. Once considered fatal in childhood, around 85% of individuals with hemoglobin SS (HbSS) now reach adulthood due to advancements in pediatric care, including newborn screening, vaccinations, penicillin prophylaxis, and treatments like hydroxyurea (5–7).

With medical advancements enabling individuals with sickle cell disease (SCD) to live longer, the transition from pediatric to adult healthcare has become a critical priority to ensure continuous, high-quality care. This shift is especially vital during adolescence a period marked by increased disease complications, risky behaviors, and heightened susceptibility to comorbid conditions, alongside other significant life changes. Healthcare transition refers to the structured, intentional process of moving young adults with chronic illnesses from child-centered to adult-oriented medical systems (1).

Despite its importance, this transition faces global challenges, including: Limited patient education about SCD and the transition process, Psychosocial barriers, Poor coordination between pediatric and adult healthcare providers, Distrust in adult care systems, and Difficulties adapting to independent disease management (8).

Without structured transition programs, patients often struggle to maintain lifelong functionality or access age-appropriate care. Many adolescents' resort to self-managing complications at home, leading to loss of follow-up, worsening comorbidities, and increased mortality (8). To mitigate these risks, national health programs must prioritize research on transition barriers to establish evidence-based best practices (9).

This study describes the proportion of patients aged 14 years and above still attending the pediatric SCC and barriers to transition from the pediatric to the adult clinic at the largest sickle cell disease clinic in Uganda

## Methods

#### Study design.

This was a mixed-method cross-sectional study with both qualitative and quantitative components. The quantitative part of the study was retrospective and used data from the year 2019 to determine the proportion of patients aged 14 years and above that attended the Mulago Hospital sickle cell clinic in that year and described their clinical and socio-demographic characteristics. The qualitative component of the study was carried out to explore the barriers to the transition of patients from pediatric to adult care.

### Study setting

The study was conducted at the Mulago National Hospital Sickle Cell Clinic (MHSCC), Kampala, Uganda. Mulago Hospital is Uganda's National Referral and teaching hospital for Makerere College of Health Sciences, Kampala. It is located on the upper Northwest of Mulago hill. It receives patients referred from health facilities within and outside Kampala District. The Department of Pediatrics and Child Health runs 6 specialized clinics, one of which is the Sickle Cell Clinic. The MHSCC has 15,500 registered patients with about 800–900 client clinic visits per month and an average of 70 to 80 patients per day, attending either for sick visits or routine follow-up. The clinic is run by pediatric haematologists, haematology fellows, medical officers, clinical officers, senior house officers, nurses, pharmacy technicians, and a few laboratory personnel. There are always about 8–12 healthcare workers at each clinic visit to render the required services.

Standard care includes: Penicillin V prophylaxis for all under-fives, Analgesia, Oral Rehydration salts (ORS) for hydration, daily Folic acid for anemia prevention, and monthly Fansidar ® for Malaria chemo-prophylaxis. Hydroxyurea is provided for free on a limited scale at the MHSCC depending on available stocks. At each visit, the attending healthcare worker does a thorough clinical evaluation including history and physical examination, and requests for laboratory investigations that include a blood smear for malaria parasites and a complete blood count. Depending on the clinician's assessment, and where required the laboratory parameters and the MHSCC protocols, the patient is managed as either an outpatient, in the day-care unit, or hospitalized at the Acute Care Unit (the pediatric emergency ward) for cases that require more monitoring and treatment.

The patients who are aged 15 years and above are given verbal communication to seek care at the adult clinic in Kiruddu Hospital or the new Mulago unit at the 4th-floor haematology clinic. Kiruddu National Referral Hospital is currently an independent National Referral Hospital Kampala city council. It is located on Buziga Hill, Makindye Division in Kampala. It's approximately 13 kilometres southeast of Mulago Hospital. The internal medicine department in Kiruddu runs many clinics including the haematology clinic that runs every Thursday 8 am-5 pm.

### Study participants

For the quantitative arm, a sample of 384 medical files of patients with confirmed and documented evidence (HB electrophoresis) of sickle cell disease aged 14 years and above who attended Mulago pediatric sickle cell clinic from January to December 2019 were selected using probability proportional to size to avoid biased sampling of patients who came early during the year. The year 2019 was used because this was a COVID-19 pre-pandemic year giving us true representation of clinic attendance. The sample size was obtained using the Kish Leslie formula for cross-sectional studies with the assumption of a 50% transition rate based on a study by Mariam Kayle et al on transition to adult care in sickle cell disease (10).

For the qualitative arm, all patients with sickle cell disease aged 14 years and above attending the pediatric sickle cell clinic of Mulago National Referral Hospital, their caregivers, and health care service providers at MHSCC who had given consent and assent, within the study period (May- July 2022) were included in the study. Patients who were critically ill and in need of urgent emergency care were excluded.

Participants of the qualitative arm were purposively selected for the interviews.

### Study procedure:

## For quantitative data collection:

A desk review of the registry was done by the principal investigator to obtain the number of patients aged 14 years and above attending the MHSCC. Upon which the proportion of patients aged 14 years and above was calculated. Then a total of 384 medical file records of patients with sickle cell disease aged 14 years and above who attended clinic from January to December 2019 were retrieved from the records office. Assuming 50% of patients were above 14 years of age, using and Leslie formula. The sample size used was 384 patients. The socio-demographic and clinical characteristics of the participants that were routinely recorded in the medical files were extracted using a pretested data extraction tool.

This data was collected concurrently with the qualitative data since one was a medical records review and the other was physical interviews of the participants attending the clinic during the study period.

### For qualitative data collection

Key informant interviews and in-depth interviews were used to obtain an in-depth understanding of the barriers to transition. Patients aged 14 years and above with SCD and their caregivers were identified and consented to the study if they fulfilled the inclusion criteria. The inclusion criteria were as follows:

- Patients above 18 years who gave consent in addition to assent for those aged 14 up to 17 years.
- Primary Caregivers of sickle cell patients aged 14 years and above attending MHSCC who had given consent.
- Healthcare providers (Head of the sickle cell clinic, haematology-oncology fellows, senior house officers, medical officers, nurses, and pharmacy technicians) at MHSCC who interacted with these patients who were present during the study period (May- July 2022) and gave consent.

Interviews were conducted in a private area that had been designated as our study space by the principal investigator assisted by a study doctor (research assistant) and one of the investigators who was well-versed in qualitative research methods. Assessment for the routine or sick visit including history taking, physical examination, and drug refills were done after completing the study interviews. The interviews were carried out every Monday, Wednesday, and Friday aside from public holidays falling on weekdays until the sample size was accrued. Each participant was assigned a study number which was appended on the patient's file, visitation card, and exercise book in addition to color coding to avoid double enrolment.

## In-depth interviews

In-depth interviews were conducted among patients aged 14 years and above attending MHSCC and their accompanying caregivers using a semi-structured interview guide to explore the barriers to transition at MHSCC. We opted for In-depth interviews because they provided free sharing space, confidentiality, and privacy for the adolescents to freely express their views. Thirty in-depth interviews were conducted and were adequate to ascertain a clear pattern. Participants were selected taking into consideration their age and gender to ensure that each category was well represented. We had 10 in-depth interviews with patients aged 14–18 years and another 10 with those aged 18 years and above attending MHSCC stratified by gender (half were males and the other half females). Ten accompanying caregivers of adolescents between ages 14–18 years were also interviewed. We were unable to interview caregivers of patients above 18 years old because almost all of them attended the clinic by themselves without an escorting caregiver. Interviews between patients and caregivers were carried out separately to provide enough room for adolescents and young adults to express their views without worrying about their caregivers. Discussions lasted at least 30–45 minutes. The interviews were held in a neutral room that allows privacy. One of the investigators who is well-grounded in qualitative research methodology guided the session. All interviews were in the language best spoken by the participant which was mostly Luganda the native language in the region and English. Interviews were tape-recorded in order not to miss any content, if participants were not comfortable with the recording, then instead notes were taken.

### Key informant interviews

A semi-structured key informant interview guide was used by a trained research assistant and the investigators to collect information on the current practices on transitioning, and health provider's perceptions towards transition of care as well as the barriers. The key informants were approached or called on the phone to schedule a convenient time for interviews by the principal investigator. Once the appropriate time was confirmed then each key informant was approached according to the schedule in areas of their convenience. Ten Interviews were conducted and each averaged 30–40 minutes. All of these interviews were conducted in English. The key informants included the head of the sickle cell clinic, two pediatric haematologists, two haematology-oncology fellows, two senior house officers, two nurses, and one pharmacy technician.

## Statistical analysis

Quantitative data was coded and double-entered into Epidata version 4.6 and then exported to STATA version 14.0 for analysis. The proportion of patients above 14 years attending the Mulago Hospital sickle cell clinic was analyzed as a frequency distribution and other categorical variables were presented in the form of frequencies and percentages.

Qualitative data from in-depth interviews and key informant interviews was analyzed using a content thematic approach. Audio recordings from in-depth interviews and key informant interviews were transcribed through repeated careful listening and generally written verbatim by the transcriber and principal investigator and then translated in text, into English before analyzing. The principal investigator identified major themes and subthemes through open coding that was done on all transcribed interviews. The development of a list of data-driven and structural codes necessitated repeated examination of the raw data. The codes were then reviewed and revised before they were grouped into categories. To ensure that they were established through reliability, a multi-step "sense-making" endeavour was used. Direct quotations were used in the presentation of results.

### Results

This was a mixed methods study carried out from May 2022 to August 2022 at the pediatric sickle cell clinic of Mulago National Referral Hospital.

By 31st December 2019, a total of 15515 patients were registered in the pediatric sickle cell clinic. However, only 4372 patients actively attended the pediatric sickle cell clinic that year (attended clinic at least twice). Of the active patients, 955 patients (21.6%) were aged 14 years and above.

## Sociodemographic characteristics of SCD patients who attended the clinic in 2019

Socio-demographic of 384 files of patients selected for the study are described. The majority of the patients, 59.4% (228/384) were aged between 14 to 18 years. There were slightly more females 53.4% (205/384) than male patients. Most of the patients (53.3%) lived within 5 kilometres of Mulago Hospital. The primary caregivers were in most cases a parent (66.6%) followed by 22.5% (86/384) who were responsible for their care. The results are summarized in Table 1 in the appendix.

Table 1
Socio-demographic characteristics of patients aged 14 and above attending the pediatric sickle cell clinic, Mulago Hospital during 2019

| Variable | Frequency | Percent (%) |
|---|---|---|
| Age (years) | | |
| 14–18 | 228 | 59.4 |
| > 18 | 156 | 40.6 |
| Sex | | |
| Male | 179 | 46.6 |
| Female | 205 | 53.4 |
| Distance from home to the sickle cell clinic. | | |
| <=5km | 214 | 53.3 |
| > 5km | 166 | 43.7 |
| Level of education | | |
| None | 62 | 19.7 |
| Primary | 105 | 33.3 |
| Secondary | 119 | 37.8 |
| Tertiary | 29 | 9.2 |
| Employment | | |
| Yes | 88 | 77.2 |
| No | 26 | 22.8 |
| Primary Caregiver | | |
| Parent | 255 | 66.6 |
| Guardian | 36 | 9.4 |
| Self | 86 | 22.5 |
| Spouse | 6 | 1.6 |

**Clinical and laboratory characteristics of SCD patients above 14 years of age in 2019.**

Clinical characteristics included haemoglobin type, SCD complications, weight, sexual development, clinic utilization, and prescribed treatments. Of 384 SCD patients who attended the sickle cell clinic in 2019, 98.2% (377/384) had HbSS. About 9.2% (35/384) had disease complications registered in their medical files and 3.4% had comorbidities. Of the 384 patients, 288 (75.4%) attended the clinic at least 4

times of which 64% (206/384 were sick visits. About 33.9% below the 5th percentile weight for age score on the CDC weight for age charts. About 48.8% of patients received hydroxyurea. Results are summarized in Table 2 in the appendix.

Table 2

**Clinical and Laboratory characteristics of patients attending the pediatric sickle cell clinic, Mulago Hospital during 2019**.

| **Variable** | **Frequency (N = 384)** | **Percent (%)** |
|---|---|---|
| Hemoglobin Type | | |
| SS | 377 | 98.2 |
| SC | 2 | 0.5 |
| Others | 5 | 1.3 |
| Clinic attendance | | |
| Once | 38 | 10 |
| 2–4 | 288 | 75.4 |
| > 4 | 56 | 14.6 |
| Routine clinic visit | | |
| Once | 131 | 40 |
| 2–4 | 186 | 56.7 |
| > 4 | 11 | 3.4 |
| Sick Visits | | |
| Once | 112 | 34.8 |
| 2—4 | 206 | 64 |
| > 4 | 4 | 1.2 |
| Comorbidity | | |
| Yes | 13 | 3.4 |
| No | 371 | 96.6 |
| Complications of disease | | |
| None | 348 | 90.9 |
| Stroke | 24 | 6.3 |
| Avascular necrosis | 6 | 1.6 |
| Leg ulcers | 5 | 1.3 |
| Sexual development/Tanner stage | | |
| stage 1 (prepubertal) | 19 | 5.9 |

| Variable | Frequency (N = 384) | Percent (%) |
|---|---|---|
| stage 2–4(developing) | 155 | 40.4 |
| stage 5(mature) | 210 | 54.6 |
| Hydroxyurea | | |
| Yes | 187 | 48.8 |
| No | 196 | 51.2 |
| PCV vaccine | | |
| Yes | 11 | 3 |
| No | 357 | 97 |
| Weight for age | | |
| < 5th percentile | 130 | 33.9 |
| > 5th percentile | 254 | 66.1 |

**Barriers to transition of care from pediatric to adult care of SCD patients attending pediatric sickle cell clinic, Mulago Hospital**

**Health system related barriers**: These were sub-divided into barriers at the pediatric sickle cell clinic and barriers at the place where the patients were to be transitioned to (adult clinic: either hematology clinic in New Mulago 4th floor or Kiruddu national referral hospital). We linked these barriers and structured them around the WHO framework that describes health systems in terms of six core components or "building blocks": (i) Good service delivery, (ii) health workforce, (iii) health information systems, (iv) access to essential medicines, vaccines and technologies (v) financing, and (vi) leadership/governance

## Leadership and Governance

## Lack of transition policy

The most frequently discussed barrier by the health care providers at the sickle cell clinic was the lack of a well written protocol to better facilitate a seamless transition from pediatric to adult health care. Health workers have tried to give verbal communication for transfer which has not always been effective. For instance, one health provider said:

*"So right now, there no developed guidelines yet, only that it was just unwritten*

*understanding that we will try to transit these patients but structurally it has not been well*

## designed, there no guiding policies that have been put in place" (KI 009)

However, some health workers also reported limited understanding on what the transition process should entail. This affected the policy making process but also the knowledge that they passed on to the patients about transition. For instance, one key informant said:

*“From your explanation of what transition is I realise now that our understanding of transition as clinicians is vague, I personally thought it means move, what have we been telling patients, my God!” (K007)*

## Access to essential medicines

## Limited access to essential drugs and drug stock outs:

Health care providers noted limited access to essential drugs and frequent drug stock outs in the adult clinic. They also received feedback from some of the patients that had attended the adult clinics about the drug shortages especially hydroxyurea, which is an essential disease modifying drug needed to improve quality of life and morphine that is largely needed for the frequently occurring pain crises

*“So, the other challenge is that medicines or drugs sometimes are not as available as they are in our clinic” (KI 009)*

## Service delivery

**Few clinic working days.**

Health care providers reported disparities in care delivery at the adult clinic such as the inconvenient clinic days because the clinic works only once a week on Thursdays and yet the nature of sickle cell disease, patients fall sick any time. As one health care provider mentioned:

*“Now for the adult clinic it runs once week in Kiruddu hospital and lower Mulago also once a week, we also have another adult clinic which runs at Uganda cancer institute also once a week so you find that the people will go but if on a day when they are not well and they find it is a day for us they will always come back to us” (KI 009)*

## Poorly structured Clinic environment

The adult clinic at New Mulago 4th floor and Kiruddu hospital are not well structured. They lack an emergency unit for resuscitation of the very sick patients, there is no clinic specific laboratory or pharmacy. Patients have to walk around the blocks to access medication or even laboratory services even when in crisis. A key informant said:

*"Yes, like I said the sickle cell clinic here is well structured, organized with a day care for emergencies and area of arrival, waiting shed, the clinical room, laboratory, pharmacy, but the adult clinic is not as well-structured you go to fourth floor and you see the doctor, the doctor sends you to the general hospital lab as opposed to the specific clinic lab" (KI 008)*

## Clinic is not sickle cell disease specific

The adult clinics both in Kiruddu and New Mulago are not sickle cell specific. The clinic reviews everyone with hematological complications which is not convenient for SCD patients. Health worker stated

*"You know it's just a hematology clinic it's not sickle cell" it's all hematology cases on that Thursday which is not convenient for the sicklers. So, if they would open up a specific sickle cell clinic for adults, I think they would move that side" (KI 001)*

## Health care work force

## Inadequate health care work force

In this study participants reported another barrier of the inadequate human resource, fair and efficient to achieve the transition process. The number of health care workers in the pediatric care working at a point in time were few to take the time to explain transition to the patients. There are very few adult carers available in adult clinics to continue the chronic care. As health care workers mentioned.

*"And then there are very few personnel/ staffs in the adult clinic at new Mulago and even*

*Kiruddu only one specialist down there it's largely the low cadre staff but here there many*

## specialists to attend to them" (KI 008)

## Poor coordination between the pediatric and adult health care providers

Health care providers expressed their grievances about the poor coordination between the pediatric and existing adult carers and the unwelcoming/poor attitude of the adult carers to work and patients. As a key informant stated:

*"We lack cooperation with those physicians, me I don't personally know any adult carer I can call to refer a patient to. They are never even available" (KI 007)*

## Individual barriers stated by patients and caregivers:

The major barriers to transition of care of SCD patients were divided into both care giver and patient factors. They reflected major concerns such as limited knowledge on transition, attachment to their pediatric carers, long distance to the adult care unit, negative experiences as shared by the patients and inappropriate age of transition by both parties.

Limited knowledge on the transition process.

Both caregivers and patients in this study expressed that their knowledge about the transition to adult health care services were limited and often absent which was more emphasized among the patients. Those that had some knowledge on the transition which was vaguely given by some health care givers did not know where the adult clinic was found and which days the clinic runs. For instance one adolescent said:

*"No, I have spent 7 years attending this clinic but I have never heard about the adult clinic and nobody has ever told me. May be when I turn 18 years there is where they said you have to sign in the file. so, I only know about that, as far as transferring from pediatric care to adult care givers I haven't heard about it" (adolescent 002*,

Similary when the caregivers were asked about their knowledge of the transition process, they simply expressed:

*"I have never heard about transition though sometimes he comes alone but he would still*

*have informed me so I have never heard about it, I have never heard that even there is*

### another clinic apart from this one" (Caregiver 008)

### Negative Experiences

Most of the adults above 18 years of age that had experienced some adult care reported bad experiences such as the unfriendly nature of the adult carers and substandard care and most of them did not want to move back, while some adolescents 14-18years and care givers had their own negative beliefs about the differences between pediatric and adult care from the stories they had heard from patients in adult care units. Some of these stories were re-echoed by some key informants whom some of the patients had confided in. For instance, one patient expressed:

"*At Kiruddu, they don't care about people, I went there when I was in pain and they did not*

*work on me, I lined up the whole day*" (*adult 004)*

Caregivers and key informants also expressed similar concerns. When interviewed they said:

*"Health workers in the adult clinic don't give us much attention and yet our children are in a*

### lot of pain" (Caregiver 006)

### Attachment to their pediatric care givers

Concerns related to leaving their pediatric providers and the peditaric environment for a new adult provider were strongly expressed. Thoughts of terminanting relationships with their pediatric carers triggered anxiety and worry for the patients and their families. These thoughts were also mirrored by their health care providers with whom patients had shared their concerns with. The patients and pediatric providers stated:

*“You really feel at home when you come at the sickle cell clinic in Mulago. I have been at Nsambya they give you the medication but then they don’t give you that touch. There is something about these people it’s like they know us and they understand us, I really appreciate them. I even feel anxious just thinking about moving” (Adult 008)*

### Discussion

A considerable number of adolescents (aged 14+) and adults with sickle cell disease (SCD) continue to rely on pediatric care services. Ensuring an effective transition to adult care is a national priority to optimize health outcomes for adolescents and young adults (AYAs) and reduce SCD-related morbidity and mortality.

This study revealed that one in four patients at Mulago Hospital’s sickle cell clinic were 14 years or older, indicating ineffective transition practices. Beyond previously recognized psychosocial and health system barriers, sociodemographic and clinical factors such as proximity to care, growth delays, and medication access also significantly hindered transition.

Key Barriers Identified were:

1. Proximity to Pediatric Care - Most patients resided within a 5-kilometer radius of the hospital, aligning with Uganda’s Universal Health Care definition of accessible distance (11). Interviews confirmed that the adult clinic’s distant location discouraged transfers, a finding consistent with Kayle et al. (2019), where AYAs nearer pediatric clinics were less likely to transition (10).
2. Delayed Growth and Development. Many patients exhibited low weight-for-age and delayed sexual maturation, leading them to perceive themselves as children rather than adults. - Caregivers and patients cited physical immaturity as a reason for staying in pediatric care, mirroring studies by Babette et al. (noted BMI declines and puberty delays) and Manuela et al. (growth retardation in SCD patients) as determinants of transition (12, 13).
3. Medication Accessibility - Hydroxyurea is consistently available at no cost in pediatric clinics but faces stockouts and fees in adult care. - Interviews revealed that AYAs returned to pediatric care due to unreliable adult clinic supplies, corroborating Morey et al.’s findings on medication access influencing transition (14).
4. Health System Challenges - Pediatric providers highlighted no formal transition protocol, adult clinic inefficiencies (e.g., limited operating days, overcrowding, and poor coordination between pediatric and adult teams). - Adult clinics lacked SCD-specific services, with fragmented layouts (e.g., separate labs/ pharmacies), complicating crisis care. Similar barriers were reported in East African HIV transition studies by Mbalinda et al and SCD research by Lebensurger et al and Wendy et al emphasizing staff shortages and poor pediatric-adult provider communication (15, 16, 17).

5. Patient and Caregiver Concerns -Limited transition awareness, emotional attachment to pediatric providers and fear of inferior adult care were major deterrents. - AYAs who attempted adult care reported long wait times, disrespectful treatment, and perceived negligence, leading some to return to pediatric clinics or disengage entirely. - These experiences align with Dimitropolous et al.'s findings on patient dissatisfaction and high dropout rates post-transition (18, 19).

### Conclusion

Multifaceted barriers spanning clinical, logistical, and emotional factors underscore the need for structured transition protocols, improved adult care services, and targeted patient education to ensure seamless, sustainable transfers for AYAs with SCD.

## Strength of the study

This study utilized both quantitative and qualitative methods of data collection which offered an extensive opportunity to describe the barriers to appropriate transition of care of SCD patients at Mulago hospital. We were able to capture the views of different stakeholders in the transition process including the patients themselves, caregivers and their health workers. This gave us a broad view of what the problem was.

The study was carried out at the Mulago pediatric sickle cell clinic which is a tertiary facility for sickle cell management in the country. Therefore, the results are indicative of what takes place in the public health facilities in Uganda with regards to transitioning patients with chronic illnesses to adult care.

## Study limitations

This being a retrospective study which involved review of patient records, it was not possible to verify information with providers or patients in real time to correct inconsistencies. However, where necessary, a phone call was made to the patient or next of kin to verify the inconsistency. Additionally, missing documentation of information such as symptoms (Vaso-occlusive episodes), measurements such as height, complications of disease and comorbidities were considered as absent and yet could have been present. This could have potentially led to misinterpretation of patient characteristics.

In this study, data was limited to only one health care system (pediatric care). We were unable to assess the views of the adult health carers on the barriers to transition of care and since most of the views by the key informants on the health care system barriers seemed to be failures in adult care system, interviewing these health workers would have helped triangulate results.

## Recommendation

The findings of this study suggest that a standardized transition approach is inadequate for this context. Tailored strategies, co-developed with patients, caregivers, and adult healthcare providers, are essential to ensure effective preparation for this process.

## Abbreviations

**ACU**
Acute Care Unit
**AYAs**
Adolescents and Young Adults
**HbSS**
Homozygous for the Sickle Cell gene
**KNRH**
Kiruddu National Referral Hospital
**MNRH**
Mulago National Referral Hospital
**MHSCC**
Mulago Hospital Sickle Cell Clinic
**SCA**
Sickle Cell Anemia
**SCC**
Sickle Cell clinic
**SCD**
Sickle Cell Disease
**SOMREC**
School of Medicine Research Ethics Committee
**UK**
United Kingdom
**USA**
United States of America
**VOC**
Vaso-Occlusive Crisis
**WHO**
World Health Organization

## Declarations

#### Ethics approval and consent to participate.

Institutional approval for this study was obtained from the School of Medicine Research and Ethics Committee (MAK-SOMREC No. 2022-285). Administrative permission was also sought from the management of MNRH. Informed consent and assent were obtained from study participants.

#### Consent for publication

Not applicable

#### Clinical trial number

Not applicable

#### Competing interest

The Authors declare that they have no competing interests

#### Funding

Research was supported by the Fogarty International Center of the National Institute of Health, U.S. Department of States Office of the U.S. Global AIDS Coordinator and Health Diplomacy (S/GAC), and Presidents Emergency Plan for AIDS Relief (PEPFAR) under Award Number 1R25TW011213. The content is solely the responsibility of the authors and does not necessarily represent the official views the National Institutes of Health.

#### Authors contributions.

The author (OR) conceived the idea and applied for funding for the research. OR, KC, GN, JK developed the proposal, supervised data collection. OR collected the data and led the manuscript writing. OR, KC, GN and JK supervised and contributed an insight to the data analysis. SK. ROO, DM, NM reviewed the manuscript. All authors contributed to the manuscript writing.

#### Availability of data and material

The original data set generated and/or analyzed during the study will be made available by the corresponding author upon request.

#### Acknowledgements

We acknowledge all the caretakers and patients at the Mulago hospital sickle cell clinic who participated in this study as well as the staff and research assistants.

#### Authors information.

[1]Department of pediatrics and child health, college of health sciences, Makerere University, Kampala, Uganda, [2]Department of Sociology, Kyambogo University, Kampala, Uganda

### Figures

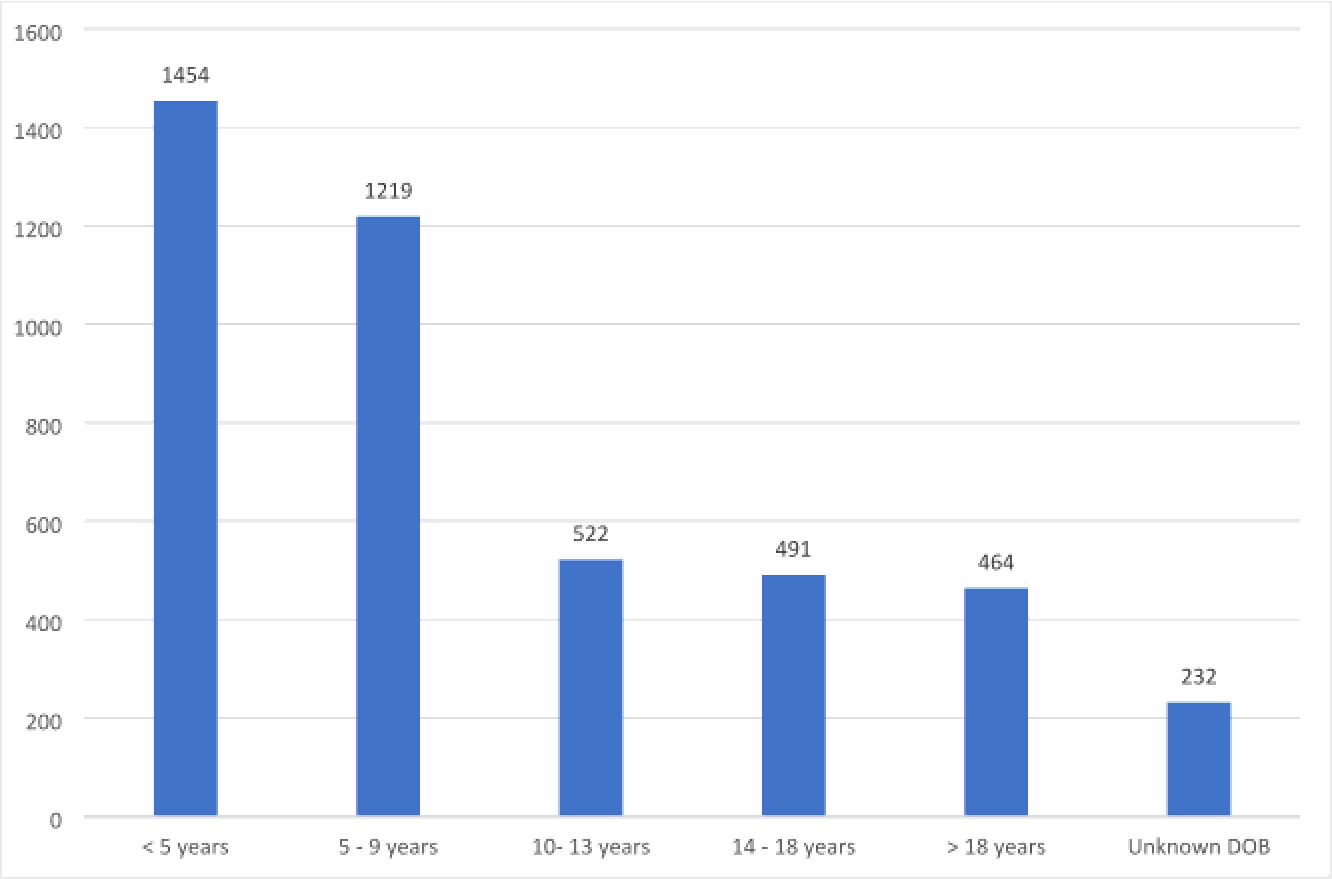

**Figure 1**

Figure 3: Bar graph showing the total number of patients distributed by age that actively attended the pediatric sickle clinic, Mulago Hospital in the year 2019.
